# Investigation of Effects and Mechanisms of Total Flavonoids of *Astragalus* and Calycosin on Human Erythroleukemia Cells

**DOI:** 10.1155/2012/209843

**Published:** 2012-06-27

**Authors:** Dongqing Zhang, Yuan Zhuang, Jichun Pan, Haibao Wang, Hui Li, Yang Yu, Deqing Wang

**Affiliations:** Department of Blood Transfusion, Chinese PLA General Hospital, Beijing 100853, China

## Abstract

Flavonoids are found in most parts of plants and have been shown to have multiple biological activities such as anticancer, anti-inflammation, antibacteria, antivirus, and immune-stimulation. Existing data showed that the total flavonoids of *Astragalus* (TFA) can provide biological system with resistance to injury and can possess antimutagenic, atherosclerotic inhibition, and other biological effects. This study investigated the effects of TFA and calycosin (a compound isolated from TFA), on apoptosis induction, and cell cycle of human erythroleukemia cell line K562 by an array of techniques, including proliferation (MTT), PI staining, Annexin V/PI double staining, and RT-PCR. The experimental data showed that TFA and calycosin could inhibit the proliferation of K562 cells. The 50% inhibiting concentrations of TFA and calycosin were 98.63 *μ* g/mL and 130.32 *μ* g/mL, respectively. However, TFA and calycosin could not induce apoptosis in K562 cells, but could increase the number of the cells in the G_0_/G_1_ phase. The level of cyclin D1 mRNA in K562 cells decreased after the treatment with TFA and calycosin. This study provides new insights into the functional mechanism of total flavonoids of *Astragalus* and calycosin on human erythroleukemia cells.

## 1. Introduction

Leukemia is a malignant tumor causing serious harm to human health, especially because of its prevalence among children [1–3]. One of the treatments being explored involves the use of total flavonoids of *Astragalus* (TFA). TFA extracted from the Mongolia *Astragalus* are the main active antioxidant ingredients for scavenging free radicals [4]. Series of studies have shown that TFA can offer biological system resistance to injury, and have antimutagenic, antitumor, inhibition of atherosclerosis and other biological effects [4–9]. Furthermore, calycosin is one of the main chemical components isolated from the TFA, and it is not clear whether calycosin can exert inhibiting influence on tumor cells or not, since there have been no reports on the mechanism for the anticancer activity of calycosin. To provide some mechanistic understanding on how TFA and calycosin affect biological system, we carry out in vitro experiments to study the antitumor activity of TFA and calycosin. We use the K562 red leukemia cell lines as the target biological system to investigate the effects and mechanisms of TFA and calycosin on tumor cells. With MTT method, we can detect the effect of different concentrations of TFA and calycosin on K562 cells and study their influences on K562 cell apoptosis, cell cycle, and cyclin D1 mRNA level. This study provides new insights into the functional mechanism of TFA and calycosin on human erythroleukemia cells.

## 2. Materials and Methods

### 2.1. Reagents

TFA and calycosin were isolated and identified by the Department of Biochemistry at the Chinese People's Liberation Army Hospital. They were sterilized by the high-pressure sterilization process, dissolved with appropriate amount of DMSO, and diluted with culture medium to the required concentrations. MTT cell proliferation and cytotoxicity assay kit, Annexin V-FITC and cell cycle kit were obtained from the Keygen Biological Technology Development. RPMI1640 culture medium and fetal calf serum were purchased from Gibco. RT-PCR extraction kit and BioEasy SYBR Green I Real-Time PCR Kit Manual were purchased from Beijing Shang Bai Company. Ribonuclease inhibitor was obtained from Biological Engineering, and M-MLV Reverse Transcriptase was purchased from Promega Biological Technology.

### 2.2. Apparatus

The following experimental apparatus were used in this study: CO_2_ Cell constant temperature incubator (Sanyo), inverted microscope (Olympus), Multiskan MK3 Microplate reader (Thermo Lab), Clean bench (Beijing East Hall Instruments), Flow Cytometry (US Backman Kurt), Line-gene Fluorescence quantitative PCR detection system (Hangzhou Rike), HH.W21-Cr Thermostatic water tank (Beijing Changan Scientific Instruments), and MS1/MS2 Mini oscillator (Guangzhou Instrument and Dental Laboratory Technology).

### 2.3. Cell Lines and Cell Culture

Human erythroleukemia cells K562 (from the Chinese PLA General Hospital hematology Lab) were cultured in the 37°C, 5% CO_2_ incubator with RPMI1640 culture solution, which contains 10% fetal calf serum. The culture solution was changed every 2-3 days. Experiments began when the cells showed a logarithmic growth.

### 2.4. Evaluation of the Effect of TFA and Calycosin on K562 Cell Proliferation

K562 cells are inoculated on a 96 orifice plate by 1 × 10^4^ cells per well, and 10 *μ*L of different concentrations of TFA and calycosin in the same medium were added to the wells. The four final concentrations of the TFA samples set are 20, 50, 100, and 200 *μ*g/mL, respectively, and the final five concentrations of the calycosin sample set are 20, 50, 100, 200, and 400 *μ*g/mL, respectively. Each concentration has six repeated wells and a blank control well with only 100 *μ*L of the RPMI1640 medium. Testing samples in the plate were cultured in an incubator and after 24 h, 20 *μ*L MTT was added to each well. After an additional 4-hour incubation at 37°C, 5% CO_2_, the supernatant was aspirated, and 150 *μ*L DMSO was added with proper mixing to terminate the reaction. The optical density (OD) value at 450 nm wavelength was measured, based on which the cancer cell growth inhibition rate was calculated. The calculation formula for the cell growth inhibition is as follows:

(1) Inhibition  (%)=(1−A450  of  Sample  GroupA450  of  Control  Group)×100%,

The calculation method for median inhibitory concentration (IC50) is based on cell survival rate on the logarithm of dose map.

### 2.5. Evaluation of the Effect of TFA and Calycosin on Apoptosis of K562 Cells by Annexin V-FITC Double-Staining Method

K562 cells, which are in the logarithmic growth phase, were inoculated in 25 mL culture bottle with 1 × 10^7^/L cell density. For both the TFA and calycosin groups and the control group (no drug), cells were collected and cultivated for 2, 4, 6, 12, and 24 h. Selection of the concentrations of TFA and calycosin is around the median inhibitory concentration. The cells were measured by flow cytometry and analysed for apoptosis population post marking the Annexin V and PI.

### 2.6. Evaluation of TFA and Calycosin Effects on K562 Cell Cycle by Flow Cytometry

Inoculate 5 × 10^6^ cells in 5 cm^2^ culture bottles; add TFA and calycosin at the concentrations of their IC50. After cultured for 24 hours, wash the cells twice with PBS, centrifuged at 2000 rpm/min for 5 min. After using PBS to blow cells into suspension completely, then fix the cells by 70% ethanol in volume at 4°C for 48 h, wash off ethanol before staining with PBS, centrifuge at 2000 rpm/min for 5 min and wash twice; add 100 *μ*L RNase A and maintain in 37°C water bath for 30 min, and then add 400 *μ*L PI staining and protect from light for 30 min at 4°C. Evaluate the cells by flow cytometric method and evaluation the changes of cell cycle. Calculate the cell proliferation index according to the formula of PI% = (S + G_2_/M)/(G_0_/G_1_ + S + G_2_/M).

### 2.7. Evaluation of TFA and Calycosin on Cyclin D1 mRNA Levels by RT-PCR

The upstream and down primers are 5-CCTCG GTGTCCTA CTTCAAAT-3 and 5-TCCTCGCACT TCTGTTCCT-3, respectively. Inoculate 4 × 10^5^/L cells in 25 cm^2^ culture bottles. Add different concentrations of TFA and calycosin, with the TFA final concentrations at 50 and 100 *μ*g/mL, and set a blank control well, incubated in the 37°C, 5% CO_2_ incubator for 48 h. Attrite cells in liquid nitrogen add 1 mg cracking liquid RL for every 50–100 mg cell mass. Use a homogenizing instrument to homogenate the cells. Sample size should not exceed 1/10 of the lysate RL volume. Apply the RT-PCR technique to detect cyclin D1 mRNA levels in K562 cells.

### 2.8. Statistical Processing

The experimental data were represented by $\bar{x}\pm s$ and analyzed by single-factor analysis of variance *t*-test using SPSS13.0 statistical analysis software. Median inhibitory concentration (IC50) was determined using probability unit method (Probit), and the cell cycle using two-sample *t*-test method.

## 3. Results

### 3.1. The Inhibition of TFA and Calycosin on Proliferation of K562 Cells

The data showed that both TFA and calycosin have significant effects on the proliferation of K562 cells after treatment for 24, 48, and 72 h (Tables 1 and 2). TFA and calycosin showed dose-dependent inhibition of K562 cells proliferation. The median inhibitory concentrations (IC50) of TFA on K562 cells after treated for 24, 48, and 72 h are 98.63, 87.90 and 63.10 *μ*g/mL, respectively. The median inhibitory concentrations (IC50) of calycosin on K562 cells after treated for 24, 48 and 72 h are 130.32, 123.03 and 122.18 *μ*g/mL, respectively.

### 3.2. The Effect of TFA and Calycosin on Apoptosis of K562 Cells

On evaluation of the experimental results of apoptotic and necrotic cells in flow cytometry, no obvious changes in apoptosis of K562 cells after treated with TFA (100 *μ*g/mL) for 2, 4, 6, 12, and 24 h were observed. Also no obvious changes in apoptosis of K562 cells after treated with calycosin (1300 *μ*g/mL) for 2, 4, 6, 12, and 24 h were observed. 

### 3.3. The Effect of TFA and Calycosin on Cell Cycle of K562 Cells

TFA (at 50 and 100 *μ*g/mL) and calycosin (at 60 and 130 *μ*g/mL) can significantly block the growth cycle of K562 cells in the stage of G_0_/G_1_ as compared with the control group. The cells in the phase of G_0_/G_1_ markedly increased and the cell in the stage of S markedly decreased (Tables 3 and 4).

### 3.4. The Effect of TFA and Calycosin on Cyclin D1 mRNA Level in K562 Cells

The cyclin D1 mRNA level was 3.58 ± 0.63 for the control sample. The cyclin D1 mRNA levels for the 50 and 100 *μ*g/mL TFA treated samples were 2.23 ± 0.42 and 1.72 ± 0.21, respectively. Similarly, the cyclin D1 mRNA level was 3.31 ± 0.71 for the control sample, the cyclin D1 mRNA levels for the 50 and 100 *μ*g/mL calycosin-treated samples were 2.27 ± 0.33 and 2.02 ± 0.25, respectively. Cyclin D1 mRNA level of K562 cells was significantly reduced after treated with either TFA or calycosin.

## 4. Discussions

Leukemia is one of the malignant tumors in hematopoietic system. It counts up to 5% in the total morbidity of cancers and with high mortality rate. K562 is the erythroleukemia cell line that is derived from human chronic myelogenous leukemia. Up to now, chemotherapy is still the most important and essential method in treating leukemia [10, 11]. The traditional chinese medicine (TCM) has made positive achievements in treating leukemia as well [12–14]. Many herbal medicines provide evident and curative effects [15–18].

*As* *tr* *ag* *al* *us* *mongholicus* Bunge is a kind of herbal medicine with a long history and extensive clinical applications. TFA is the active component isolated from *Astragalus* with peroxyl radical scavenging capacity [4]. Studies showed that TFA have anti-injure and antimutation activities [4, 5, 7, 8]. Furthermore, TFA have a significant inhibitory effect on human hepatocellular carcinoma BEL-7402 cells in vitro [6].

Because of the difference in DNA content, the cell cycle is divided into discrete phases: G_0_/G_1_, S, and G_2_/M. Based on the experimental results, we calculate the distribution percentage of the studied cells in each phase using a software. It is known that tumor cells in the S phase is higher than normal cells [19], and we also observed a large number of cells are in S phase with active DNA synthesis. The experimental results further show that TFA causes the cells in the G_0_/G_1_ phase to increase, particularly cells in the G_1_ phase increased, and the cells in the S stage were significantly reduced, which indicated that the proliferative activity of K562 cells had weakened upon treatment.

Cyclin D is a sensor for extracellular growth signals. The existence of growth factors lead to continuous expression of cyclin D. Cyclin D1 begins to express between the G_0_ and G_1_ phases and participates in the control of the G_1_ phase by binding to cyclin-dependent kinases 4 (CDK4) and 6 (CDK6), leading to the progression of the cells into the S phase to proliferate. Deregulation of the cell cycle will be induced by uncontrollable expression of cyclin D1 or CDK4/CDK6. Studies indicate that many tumors have overexpression of cyclin D1, such as mantle cell lymphoma (CML), nonsmall cell lung cancer (NSCLC), breast cancer, head and neck cancer, and esophageal cancer [20, 21].

Cyclin D1 is also an important regulatory protein for cell cycle. The level and activity of cyclin D1 reach to the peak at different phases with regular wave. During G_0_, the cell is subjected to stimulation by extracellular mitogens and subsequently expresses cyclin D1 in the early part of G_1_. Cyclin D1 participates in the control of the G_1_ phase by binding to CDK4/CDK6. Cyclin D1-CDK4 complexes phosphorylate retinoblastoma1 (RB) by the array of LXCXE at its N-terminal. Upon RB phosphorylation by CDK4/6, RB dissociates from E2F-DP1 heterodimer, leading to the inactivation of its suppressor effect on DNA synthesis and the subsequent progression of the cell into S phase from G_1_ [22]. There are few studies on the effects of cyclin D1 on leukemia either domestically or abroad. In this study, we find that TFA and calycosin can notably reduce the expression of cyclin D1, which is likely related to the effects of TFA and calycosin in suppressing the propagation of K562 and retaining more cells in G_0_/G_1_ phase.

 In conclusion, TFA and calycosin had effects on inhibition of the proliferation of K562 cells. They are also attributed to arrest them in the G_0_/G_1_ phase and induce decreased cyclin D1 mRNA.

## Figures and Tables

**Table 1:** The inhibition of TFA on proliferation of K562 cells.

TFA concentration (*μ*g/mL)	24 h		48 h		72 h	
	OD value	Inhibition rate %	OD value	Inhibition rate %	OD value	Inhibition rate %
Control group	0.49 ± 0.02	0.5	0.59 ± 0.02	0.6	0.62 ± 0.04**	0.7
20	0.46 ± 0.03	5.8	0.46 ± 0.04**	22.7	0.48 ± 0.01**	23.5
50	0.42 ± 0.04**	14.9	0.40 ± 0.03**	31.4	0.32 ± 0.01**	48.9
100	0.28 ± 0.04**	44.1	0.34 ± 0.06**	42.8	0.24 ± 0.01**	62.4
200	0.06 ± 0.03**	88.5	0.16 ± 0.04**	72.9	0.09 ± 0.02**	85.0

Data expressed as mean $\bar{x}\pm s$ , **P < 0.01 compared with control group.

**Table 2:** The inhibition of calycosin on proliferation of K562 cells.

Calycosin concentration (*μ*g/mL)	24 h		48 h		72 h	
	OD value	Inhibition rate %	OD value	Inhibition rate %	OD value	Inhibition rate %
Control group	0.48 ± 0.01	0.6	0.56 ± 0.02	0.1	0.70 ± 0.02**	0.01
20	0.41 ± 0.02	15.1	0.49 ± 0.03**	18.8	0.56 ± 0.02**	19.7
50	0.34 ± 0.02**	30.2	0.41 ± 0.05**	31.2	0.48 ± 0.01**	30.4
100	0.27 ± 0.01**	45.0	0.34 ± 0.02**	43.2	0.39 ± 0.03**	43.3
200	0.21 ± 0.02**	57.9	0.25 ± 0.01**	58.1	0.29 ± 0.01**	57.9
400	0.15 ± 0.01**	67.9	0.17 ± 0.01**	71.2	0.19 ± 0.01**	72.0

Data expressed as mean $\bar{x}\pm s$ , **P < 0.01 compared with control group.

**Table 3:** The effect of TFA on cell cycle of K562 cells.

Groups	G_0_/G_1_ stage (%)	S stage (%)
Control group	31.79 ± 2.98	63.59 ± 2.47
TFA (50 *μ*g/mL)	36.32 ± 2.56	55.41 ± 1.65**
TFA (100 *μ*g/mL)	47.52 ± 1.73**	52.82 ± 1.33**

Data expressed as mean $\bar{x}\pm s$ , **P < 0.01 compared with control group.

**Table 4:** The effect of calycosin on cell cycle of K562 cells.

Groups	G_0_/G_1_ Stage (%)	S Stage (%)
Control group	31.61 ± 2.67	70.09 ± 2.08
Calycosin (60 *μ*g/mL)	40.89 ± 2.56	57.47 ± 1.89**
Calycosin (130 *μ*g/mL)	46.33 ± 2.88**	54.79 ± 1.94**

Data expressed as mean $\bar{x}\pm s$ , **P < 0.01 compared with control group.
